# Neurofeedback Therapy for Sensory Over-Responsiveness—A Feasibility Study

**DOI:** 10.3390/s22051845

**Published:** 2022-02-25

**Authors:** Ruba Hamed, Limor Mizrachi, Yelena Granovsky, Gil Issachar, Shlomit Yuval-Greenberg, Tami Bar-Shalita

**Affiliations:** 1 Department of Occupational Therapy, Faculty of Medicine, School of Health Professions, Tel Aviv University, Tel Aviv 6997801, Israel; ruba.mjh@gmail.com (R.H.); limor092@gmail.com (L.M.); 2 Laboratory of Clinical Neurophysiology, Department of Neurology, Faculty of Medicine, Rambam Health Care Campus, Technion, Haifa 3109601, Israel; y_granovsky@rambam.health.gov.il; 3 Biomedical Engineering Department, Faculty of Engineering, Tel Aviv University, Tel Aviv 6997801, Israel; gil.issachar@gmail.com; 4 School of Psychological Sciences, Tel Aviv University, Tel Aviv 6997801, Israel; shlomitgr@tau.ac.il; 5 Sagol School of Neuroscience, Tel Aviv University, Tel Aviv 6997801, Israel

**Keywords:** sensory processing, sensory modulation dysfunction, neuromodulation, neurofeedback, pain sensitivity, life satisfaction, goal attainment

## Abstract

Background: Difficulty in modulating multisensory input, specifically the sensory over-responsive (SOR) type, is linked to pain hypersensitivity and anxiety, impacting daily function and quality of life in children and adults. Reduced cortical activity recorded under resting state has been reported, suggestive of neuromodulation as a potential therapeutic modality. This feasibility study aimed to explore neurofeedback intervention in SOR. Methods: Healthy women with SOR (n = 10) underwent an experimental feasibility study comprising four measurement time points (T1—baseline; T2—preintervention; T3—postintervention; T4—follow-up). Outcome measures included resting-state EEG recording, in addition to behavioral assessments of life satisfaction, attaining functional goals, pain sensitivity, and anxiety. Intervention targeted the upregulation of alpha oscillatory power over ten sessions. Results: No changes were detected in all measures between T1 and T2. Exploring the changes in brain activity between T2 and T4 revealed power enhancement in delta, theta, beta, and gamma oscillatory bands, detected in the frontal region (*p* = 0.03–<0.001; Cohen’s *d* = 0.637–1.126) but not in alpha oscillations. Furthermore, a large effect was found in enhancing life satisfaction and goal attainment (Cohen’s *d* = 1.18; 1.04, respectively), and reduced pain sensitivity and anxiety trait (Cohen’s *d* = 0.70). Conclusion: This is the first study demonstrating the feasibility of neurofeedback intervention in SOR.

## 1. Introduction

Sensory over-responsiveness (SOR), a sensory processing neurodevelopmental alteration, affects the ability to regulate adaptive responses to sensory stimulation, in single or multiple sensory modalities [1,2,3,4]. SOR is characterized by an augmented intensity, longer duration, or painful patterns of response to non-noxious sensations [2]. Additionally, testing daily pain revealed enhanced pain sensitivity in individuals with SOR compared with normoresponsive people [5,6]. Furthermore, utilizing experimental pain via psychophysical testing indicated hyperalgesia (amplified pain intensity) and lingering pain sensation [7,8,9,10], as well as altered physiological reactivity [11,12], which may suggest compromised endogenous pain modulation in otherwise healthy children and adults with SOR [10,13]. Moreover, SOR has been widely reported to be associated with psychological distress and emotionality, e.g., [14,15,16], and both are also considered to be factors in phenotyping pain [17]. Indeed, SOR greatly interferes with well-being, daily function, and quality of life [5,14,16,18,19], and while the estimated incidence reported among typical children and adults is 5–16% [5,20,21], effective therapy, supported in evidence-based practice for adults with SOR, is scarce.

Brain regions identified as the “pain matrix” are equally involved in processing painful and non-painful stimuli, emphasizing the interwoven pain and sensory processing mechanisms [13,22], and may elucidate the co-occurrence of SOR and amplified pain sensitivity [23,24,25]. Indeed, enhanced responses to sensory stimuli have been reported in chronic pain syndromes, such as fibromyalgia [26,27,28,29] and migraine [30,31,32,33], and have been suggested as a contributing factor for chronic pain development [22,34]. These findings may indicate that, like pain, SOR is associated with thalamo–cortical dysrhythmia [35,36]. Therefore, the association between pain and SOR may be related to the anatomical integration of sensory and pain transmitting pathways in the thalamic nuclei that project to cortical areas involved in the perception of painful and non-painful stimuli [37]. Amplified sensory painful and non-painful sensitivity is mirrored in the cortical activity and can be measured by the electrical oscillations of the neural activity using electroencephalogram (EEG) [38]. In experimental pain studies using EEG resting-state neural recording, the most explored EEG signal is the alpha band. Alpha is considered an inhibitory oscillation [39], and in pain conditions shows reduced power [40,41,42,43]. Like pain patients [44,45,46,47], individuals with SOR during EEG resting-state recording demonstrated an overall reduction in cortical activity, most prominently in the alpha band power [48]. Interestingly, alpha activity within the sensory cortex is linked to environmental stimulation intake, and lower power is suggested to accompany a state of higher stress or arousal, both characterizing individuals with SOR [14,16,48]. Since identifying factors contributing to the pain experience can direct the management of the underlying pathophysiology [17], SOR may serve as a predisposition [49], and, as such, can be targeted in preventing pain development or chronification [13], utilizing neuromodulation. Neurofeedback is a non-invasive and non-pharmacological neuromodulatory intervention aimed to ameliorate cortical activity [50].

EEG-based neurofeedback therapy, a neuromodulation technique [51], is an operant learning biofeedback during real-time acquisition of EEG recording at rest, and is based on the endogenous brain neuroplasticity, designed to elicit a long-lasting self-regulation of the brain’s altered neural activity [52,53]. Through the EEG biofeedback, individuals learn to self-regulate their brain oscillatory activity and affect their central nervous system, in order to practice recognition of mental strategies linked to brain states which are related to therapeutic gains [54]. Indeed, recent studies, e.g., [55], and systematic reviews [53,56] have shown the utility of neurofeedback therapy to obtain significant pain reduction lasting several months, e.g., [45,46,57], as well as its advantage over pharmacological [45] and non-pharmacological [58,59] analgesic treatments. However, EEG-based neurofeedback has not yet been studied in individuals with SOR. This feasibility study aimed to gain insight on whether neurofeedback therapy is a potential tool for ameliorating brain activity at rest and symptom severity in otherwise healthy adults with SOR. We hypothesized that neurofeedback therapy would positively impact both neurophysiological (i.e., upregulating alpha oscillation power) and behavioral (i.e., enhanced life satisfaction, reduced pain sensitivity and anxiety, and personalized treatment goals achievement) outcomes, and induce long-term effects.

## 2. Materials and Methods

**Figure 1:**
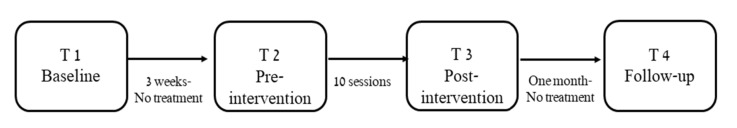
Study design.

This experimental feasibility study included 4 measurement time points (T1–4) (Figure 1): T1 (baseline) served as control for T2 (preintervention), T3 (post-intervention), and T4 (follow-up) to test the therapeutic effect over time. The assessor who performed the 4 measurements differed from the researcher who conducted the neurofeedback sessions. See Appendix A for information defining this study according to the consensus on the reporting and experimental design of clinical and cognitive–behavioral neurofeedback studies (CRED-nf checklist), a checklist assuring best practice in neurofeedback [51].

The study was approved by the Institutional Review Board, was and registered in the Clinical Trials Protocol Registration and Results System (PRS) (#1710.18). All participants provided signed informed consent.

### 2.1. Participants

Healthy women with SOR aged 21–50 years were recruited via a convenience sampling utilizing social networks, and the snowball method. Adhering to the reported sex variance in brain activity [60] and cortical neuroplasticity [61], this study tested females only. Inclusion criteria stipulated a score higher than 2.39 on the sensory responsiveness questionnaire, aversive scale, indicating SOR, free of analgesic for 24 h before study sessions, and adequate language skills. Exclusion criteria included: metabolic, psychiatric, neurological, or neurodevelopmental diagnoses; acute or chronic pain; regular intake of neurological, psychiatric and analgesic medicines; currently participating in other non-pharmacological therapies (e.g., cognitive therapies) that commenced within the past 6 months; substance abuse (e.g., more than 10 glasses of alcohol per week [62,63]; pregnancy, or breast-feeding. Participants were requested to commit to attending 80% of the treatment sessions and to avoid the use of substances up to 6 h prior to sessions [64,65]. Since no reports testing neurofeedback in SOR were found, the sample size was determined based on neurofeedback studies in participants with ADHD [66,67], a comorbid condition with SOR [68,69,70,71].

### 2.2. Instrumentation

#### 2.2.1. Screening Measure

Sensory responsiveness questionnaire—Intensity Scale (SRQ-IS) [72]: A self-report questionnaire, which aims to clinically identify sensory modulation dysfunction in adults. The SRQ-IS contains a set of 58 items of daily life scenarios that are presented in an aversive/hedonic manner. Each scenario entails one sensory stimulus in one modality, including auditory, visual, gustatory, olfactory, vestibular, and somatosensory, except for pain. Participants are asked to rate the intensity of the sensory responses to each scenario using a 5-point scale ranging from 1 (not at all) to 5 (very much) and yielding 2 scores: SRQ aversive and SRQ hedonic. Scoring higher than the mean +2SD for at least one of both SRQ scores indicates sensory modulation dysfunction (SRQ aversive: 1.87 + 0.52 indicating SOR; and SRQ hedonic: 2.10 + 0.66; indicating sensory under-responsivity), e.g., [48]. The SRQ was reported to have content, construct, and criterion validity, internal consistency (Cronbach’s α = 0.90–0.93), and test–retest reliability (r = 0.71–0.84; *p* < 0.001–0.005) [72]. In this study, SOR was identified by applying the SRQ aversive sub-scale score >2.39 (32 items).

#### 2.2.2. Primary Outcome Measure

Resting state (EEG) recording: A 5 min continuous EEG recording, using a 64-channel cap (Quik-Cap SynAmps, Compumedics Neuroscan) and an EEG machine (Curry 7 EEG system, Compumedics Neuroscan), was performed during relaxed wakefulness with eyes closed [73]. Additional parameters included a bandpass filter from 0.1 to 100 Hz, a 500 Hz sampling rate, an electrode impedance less than 5 kOhm, and a notch filter of 50 Hz to reduce electrical interference.

EEG data processing: EEG recordings were analyzed with Curry 7 Software (Compumedics Neuroscan). The data was re-referenced to the common average of all electrodes. The raw data was band passed filtered to the range 0.5–45 Hz. Next, each raw signal was visually inspected to detect extremely noisy intervals, which were removed from further analysis. Removed intervals were replaced with a linear interpolation between the noisy interval’s edges. Later, blinks and eye movements artifacts were further removed by automatic detecting and reducing artifactual independent components. The automatic detection of artifacts was based on running independent component analysis (ICA) and comparison to vertical electrooculogram (VEO) channel extreme amplitudes (the threshold was tuned by visual inspection for each subject).

To extract power spectrum measures: Each 5-minute recording section was divided into 300 segments of 1 s. Power spectral densities were computed by averaging the fast Fourier transformation power spectra of each 1 s segment. We used the average power in each frequency band including delta (1–4 Hz), theta (4–8 Hz), alpha (8–12 Hz), beta (12–30 Hz), and gamma (>30 Hz) bands [74].

#### 2.2.3. Secondary Outcome Measures

Satisfaction with Life Scale (SWLS) [75]: A self-report questionnaire which assesses global life satisfaction, and examines the cognitive component of subjective well-being [76]. By using a scale ranging from 1 (strongly disagree) to 7 (strongly agree), participants were asked to rate their level of agreement with 5 statements reflecting the overall satisfaction with one’s life. The final score is the 5-statement sum, ranging from 5 (minimum life satisfaction), to 35 (high life satisfaction). Cronbach’s α of 0.87 and a 2-month test–retest stability coefficient of 0.82 were reported.

Pain Sensitivity Questionnaire (PSQ) [77]: A self-report questionnaire which assesses daily somatosensory pain sensitivity. Comprising 17 items and utilizing a scale ranging from 0 (not painful at all) to 10 (worst pain imaginable), participants are asked to rate imagined painful daily life situations. Fourteen of the items describe painful situations for the majority of people (e.g., hot, cold, sharp, and blunt). The other 3 items (items 5, 9, 13) describe normally non-painful situations. The PSQ provides a total score and two subscale scores: PSQ moderate and PSQ minor. The PSQ has been demonstrated to have content, criterion, and construct validity, as well as internal consistency (Cronbach’s α = 0.92 for PSQ total, 0.81 for PSQ minor, and 0.91 for PSQ moderate), and test–retest reliability (ICCs = 0.83, 0.86, and 0.79, respectively).

State–Trait Anxiety Inventory (STAI) [78]: A self-report questionnaire which aims at assessing anxiety utilizing 2 parts: (i) the anxiety felt currently (state), and (ii) the generally felt anxiety (trait). Each part consists of 20 items. Participants were required to rate the level of anxiety using a 4-point scale ranging from 1 (Almost Never) to 4 (Almost Always); higher scores indicate more anxiety. The STAI was demonstrated to have internal consistency (Cronbach’s α = 0.86), test–retest, and high intraclass correlation coefficient.

Goal Attainment Scaling (GAS) [79]: A standardized method which is used to evaluate the participant’s progress toward their functional goals. Participants specified two goals that demonstrated specific, measurable, acceptable, relevant, and time-related (SMART) components. A 5-point scale (from –2 to +2) is used for scoring the change towards goal attainment. Zero indicates the expected level of performance; −2 indicates much less than the expected performance (reported as baseline in this study); −1 indicates somewhat less than expected performance; +1 indicates somewhat more than the expected performance; and +2 indicates much more than the expected performance. The GAS has been found to be significantly effective in identifying meaningful outcomes in families of children with SMD [80]. Good reliability [81], as well as satisfactory interrater reliability (r = 0.51–0.91), responsiveness, content, and convergent validity, were reported [82,83].

#### 2.2.4. Intervention

Neurofeedback (NF): Applying EEG (Curry 7 EEG system, Compumedics Neuroscan) and an auditory feedback module [84], the treatment system trains adults with SOR to normalize (i.e., work towards enhancing the value) the alpha band power (amplitude). Apart from the EEG machine, the apparatus includes the recording electrodes mounted to the Quik-Cap SynAmps, Compumedics Neuroscan, and two PC screens—(i) the therapist feedback screen used for tracking the participant performance, and (ii) the control panel screen which displays the EEG recording. Via ATH-M50x earphones, each treatment session started with 1 min resting-state recordings (adaptation phase), that provided real-time alpha power measure which the system automatically used as the baseline. Following the system’s automatic adjustment [84], participants were engaged in 19 min of NF treatment (training phase), which included listening to babbling brook sounds (the feedback), which became louder linearly with the alpha power elevation [85]. A 1 min break after every 3 min training was provided. In each session, standardized instructions were given to close their eyes [73], relax deeply without falling asleep, and to continuously try to maximize the volume of the sound as much as possible; information on how to gain control of brain activity was not provided [73]. This neurofeedback system has been programmed to support learning processes by implementing the following principals: (i) upregulated alpha was demonstrated by the automatic scoring calculation, which the neurofeedback system presents at the end of each session. Specifically, the baseline level of each subject was calculated automatically and the distance between the baseline level and the desired target level (+5%) was automatically set at the beginning of each session. During the training session, whenever reaching the target, the subject gains a point. If the subject succeeds in keeping brain activity in the targeted zone for a targeted duration, then they earn extra points, equal to the number of samples in the sequence [84]. Thus, if alpha upregulation does not occur, no scoring is presented. (ii) Following positive feedback, the software pauses the feedback display for 1 sec, allowing the participant time to internalize and learn from the successful process [84].

### 2.3. Procedure

After verifying inclusion criteria, the GAS followed by the neurophysiological assessment were performed onsite. The PSQ, SWLS, and STAI were completed online using a “Google Forms” platform in two different questionnaire sequences, ruling out possible fatigue or concentration effects. These measures comprised each of the 4-measurement time points, which were scheduled as follows: T2 and T4 were conducted three weeks post-T1, and four weeks post-T3, respectively. Treatment started after T2 and included 10 therapy sessions [85,86] of 45 min each, held at the same time of day, twice a week (with a maximum of 5 days between sessions). Participants were seated in a reclining position facing a white wall. T3 was performed immediately after the 10-session intervention terminated.

### 2.4. Statistical Analyses

Data was analyzed using the Statistical Package for the Social Sciences Software (SPSS) version 24. Study measures were summarized via descriptive statistics by data type. Normal distribution was tested using Shapiro–Wilks test. Using Mann–Whitney, score differences between the 2 questionnaire sequences were tested. Wilcoxon matched–paired signed rank test (secondary outcome measures) or repeated measure ANOVA (primary outcomes) were used for analyzing the dependent variables change over time. Using two-tailed cutoff a *p*-value has been set at 0.05. Effect size for clinical meaningfulness between T2 and T4 was calculated via Cohen’s d: 0.2—small effect; 0.5—medium effect; 0.8—large effect; >1—very large effect. T2 and T4 were chosen to test the clinical meaningfulness, since follow-up findings indicated the neurofeedback effect [53].

## 3. Results

### 3.1. Sample Dropout

**Figure 2:**
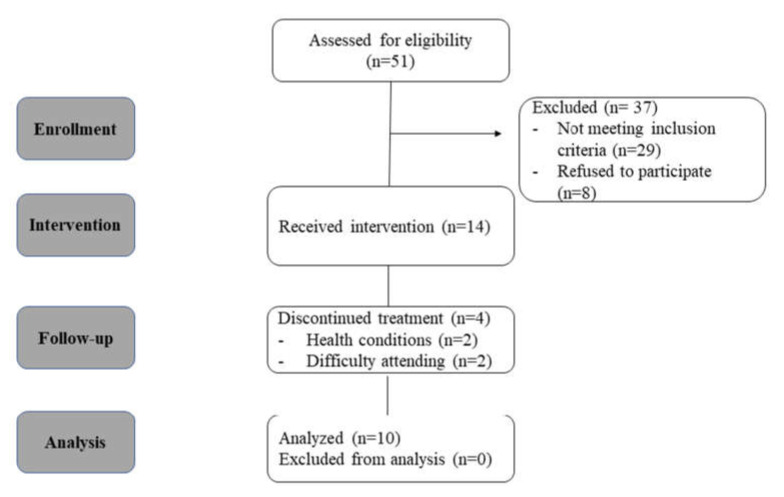
Recruitment and study participants diagram.

During 10 months (December 2018–September 2019), 51 potential participants approached via mail, phone, or social networks. A total of 37 participants (72.5%) were excluded: 29 did not meet the inclusion criteria and 8 dropped out before starting (due to distant study location). Retention was 14 females (27.5%), 4 of the 14 participants (29%) dropped out during the intervention process due to health conditions or difficulty attending the meetings (due to distant study location), and 10 (71%)—age range 27–47 years (Table 1)—completed the treatment (See Figure 2).

### 3.2. Upregulating Alpha during Training

**Figure 3:**
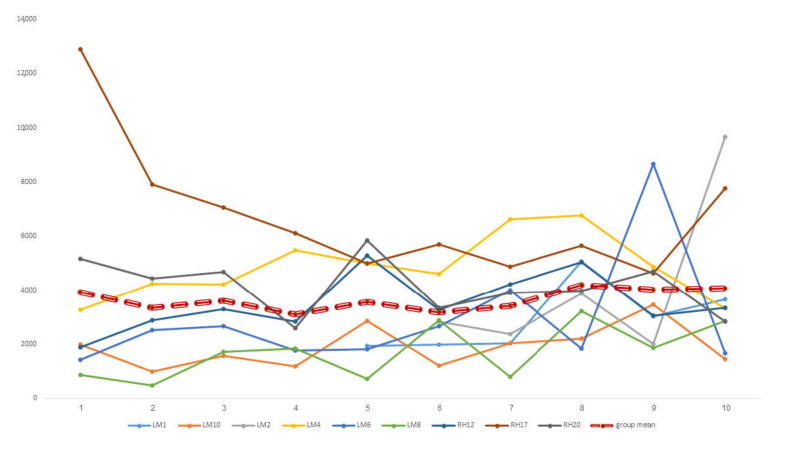
Upregulating alpha automatic scoring for each participant during each of the ten sessions, and the group mean (bold dotted line). Note: missing data for one subject; additionally, two participants first succeeded upregulating alpha only at sessions 5/6.

Most of the participants managed to upregulate their alpha activity, as reflected in their session scores (Figure 3).

### 3.3. Primary Outcomes Measure

Neurofeedback and resting state EEG: Applying repeated measures ANOVA on the EEG alpha band responses, we found no statistically significant change over time in alpha band power (Table 2).

Using Lim et al.’s (2018) method for tracking brain regions via EEG electrode recording we found statistically significant change over time in delta, theta, beta, and gamma power only in the frontal region (Table 2), indicating higher power at T4 (*p* = 0.03–0.001). Post hoc analyses adjusted for multiple comparisons indicated statistically significant change only at T4 in *delta* and *theta* bands power (Table 2).

Testing effect sizes between T2 and T4, we found sub-large–very large effect sizes. A very large effect size was found in the *theta* power recorded at frontal electrodes (*d* = 1.13) (Table 2). Testing the effect size between T2 and T3, we found a moderate effect size in theta, and a small effect size in the other bands tested (see Table 2).

### 3.4. Secondary Outcome Measures

Descriptive statistics of behavioral measures—SWLS, GAS, PSQ, and STAI (mean, SD, median, and interquartile range (IQR))—across the 4 measurement time points (T1—T4) are presented in Table 3.

No significant differences between the two questionnaire sequences at T1 were found (*p* > 0.05).

#### 3.4.1. Neurofeedback and Life Satisfaction

No statistically significant difference between T2 and T3 in the SWLS scores was found (*p* = 0.09). However, examining the magnitude of the treatment effect on the SWLS scores between T2 and T4, we found a very large effect size (*d* = 1.18) and a statistically significant difference (*p* = 0.016). Furthermore, we found no statistically significant differences in the SWLS scores between T1 (baseline) and T2 (*p* = 0.51) or between T3 and T4 (*p* = 0.41).

#### 3.4.2. Neurofeedback and Pain Sensitivity

Testing the differences between T2 and T3 in the PSQ scores, we found a statistically significant difference in the PSQ total (*p* = 0.047), a statistical trend in the PSQ minor (*p* = 0.06), and a non-significant difference in the PSQ moderate (*p* = 0.13) scores.

Examining the magnitude of the treatment effect (T2 and T4) in the PSQ scores, we found sub-large effect size at both PSQ total and minor sub-scale scores (*d* = 0.70, 0.73, respectively), and medium effect size in the PSQ moderate sub-scale (*d* = 0.50). No statistically significant differences in the PSQ (total, minor, moderate) scores between T1 and T2 (*p* = 0.26, 0.78, 0.10, respectively), or between T3 and T4 (*p* = 0.72, 0.77, 0.92, respectively) were found.

#### 3.4.3. Neurofeedback and Anxiety

No statistically significant differences in STAI sub-scale scores (state, trait) between T2 and T3 were found (*p* = 0.36, 0.14, respectively). However, examining the magnitude of the treatment (T2 and T4), we found sub-large effect size in the *trait* sub-scale (*d* = 0.70) but not in the state sub-scale (*d* = 0.02).

No statistically significant differences in the STAI sub-scale scores between T1 and T2 (*p* = 0.63, 0.48, respectively) or between T3 and T4 (*p* = 0.68, 0.62, respectively) were found.

#### 3.4.4. Neurofeedback and Achieving Personalized Goals

A statistically significant difference between T2 and T3 in the GAS scores (*p* = 0.012) was found. Moreover, examining the magnitude of the treatment effect in the GAS scores between T2 and T4, we found a very large effect size (d = 1.04) and a statistically significant difference (*p* = 0.015). No statistically significant differences between the GAS scores in T1 and T2 (*p* = 0.18) and in T3 and T4 (*p* = 0.46) were found.

### 3.5. Correlations between Primary and Secondary Outcomes

Testing the correlations between the primary and secondary outcome measures changes, calculated as the deltas between T4 and T2 for each outcome measure, we found statistically significant correlations only between *delta band* power and the GAS scores (Spearman’s ρ = 0.81, *p* = 0.015), as well as in the *pick alpha frequency* and the PSQ total score (Spearman’s ρ = 0.77, *p* = 0.026).

## 4. Discussion

According to the consensus on the reporting and experimental design of clinical and cognitive–behavioral neurofeedback studies (CRED-nf checklist) recently published [51], an ideal neurofeedback effectiveness should be evident in the targeted brain activity change intra- and inter-sessions, and in comparison with a control group for negating other non-specific effects [51]. The alpha power did not change during the course of this study and a control group was not tested. However, “Feasibility studies are pieces of research done before a main study in order to answer a question: Can this study be done? as well as (i) to estimate important parameters that are needed to design the main study” [87] and (ii) to reveal preliminary responses to intervention [88]. Thus, this study aimed at a feasibility study testing neuromodulation via neurofeedback in women with SOR. To the best of our knowledge, this is the first report studying neuromodulation in SOR, a health condition that not only severely interferes with everyday function and quality of life [5,14,16,18,19], but also posits a risk factor in developing chronic pain [22,34]—yet, interventions for alleviating SOR effects in adults are sparse. While targeting the alpha activity, this study found no change in the alpha band activity. However, changes were detected in the other oscillatory bands. Importantly, the changes in oscillatory power occurred only at follow-up testing, indicating a long-term effect [52]. Examining the behavioral measures revealed improved scores at follow-up, and since no differences were detected between T1 and T2 (both measured before intervention), as well as between T3 and T4 (measured after intervention), we believe that our findings may indicate that neurofeedback-related changes and their stability last one month post-treatment. Of note, since follow-up measurements are the ones indicating the neurofeedback effect [51,53], and indicating whether neuroplasticity was induced [52], we determined the neurofeedback impact based on the changes occurring between pretreatment and follow-up. Furthermore, presenting the findings in respect to *p* value, as well as to clinically meaningfulness analyses, allowed us to better explore the treatment gains [89,90]. Indeed, along the significantly increased power in frontal electrodes mentioned above, testing the clinical meaningfulness of change (i.e., the notion that a change is of everyday importance [89]), we also revealed moderate to very large effect size achieved via this treatment approach in otherwise healthy individuals with SOR. Moreover, we found sub-large effect size in reducing pain hypersensitivity and anxiety trait, a very large effect size in achieving personalized goals, and a very large effect size in enhancing life satisfaction.

Several studies have attempted to alleviate pain in chronic pain patients by targeting the upregulation of alpha power in isolation or combined with other bands via neurofeedback [46,56,91]. Interestingly, while indicating pain reduction following neurofeedback, very few demonstrated an increase in alpha power [56], in line with our findings showing a lack of change in alpha oscillations post-neurofeedback. Yet, the pick alpha frequency—A well-recognized marker for pain sensitivity [92,93] utilized as a target for alleviating pain, e.g., [44]—was found to be linked to self-reported daily pain sensitivity in this study. This finding supports previously reported coupling between alpha oscillations and pain sensitivity [44,46,47]. However, recently, it was suggested that alpha power may not measure the success of learning underlying pain reduction via neurofeedback. Specifically, alpha oscillation is characterized by two modes of high and low amplitude [52], suggesting that extracting the mean power may be misleading [47]. Indeed, parameters measuring the dynamics in alpha state, not reflected in the mean power, were found as more sensitive to capture learning [47]. However, we found a very large effect size, indicating meaningful changes in delta and theta bands. Indeed delta and theta oscillatory bands are mutually connected to alpha, such that alpha is related to inhibition of behavioral patterns linked to delta and theta activity, and is employed via the prefrontal cortex [94]. Specifically, alpha power oscillations arise in the thalamo–cortical feedback loop, partly generated in the anterior brain regions [95], and are linked to expectancy, memory, and cognitive processes [96,97,98,99]. The neurophysiological underlying mechanism for delta, theta, and alpha oscillation reciprocity is unknown; yet it has been hypothesized to be mediated by the prefrontal cortex, balancing cognitive functions linked to alpha, and evolve as an integrated and efficient organized-behavior-oriented mechanism, achieving important goals [94]. Indeed, in this study, the EEG-related effect of neuromodulation occurred only in the frontal region, which underlies cognition, and interestingly, we found a very large effect size in achieving personalized goals. The latter change from pretreatment to follow-up was found correlated only with delta band change. Since low cortical or behavioral arousal is coupled with significant low-frequency oscillations [50], and since individuals with SOR are characterized by high arousal [48], our findings suggest that neuroplasticity achieved via neurofeedback attenuated arousal, mirrored in delta oscillation, which may have enabled proceeding toward personalized goal attainment. Thus these findings may support the feasibility of neurofeedback in individuals with SOR.

Behaviorally, this study demonstrates an increase in life satisfaction—regarded as the cognitive component of well-being [100]. Life satisfaction was enhanced from neutral (i.e., equally satisfied and dissatisfied) in T2 to slightly satisfied post-NF (T3) therapy and follow-up (T4). Thus, though not at post-intervention, findings reveal a very large effect size and statistically significant change at follow-up (T4), attaining a life satisfaction level which agrees with the average satisfaction reported in the Western population [101]. As for state anxiety, finding no effect is in line with previous reports [86]. However, demonstrating treatment efficacy in trait anxiety only at a one-month follow-up may be explained by brain modification, which has been linked to a long-term decrease in anxiety symptoms [102]. Importantly, long-term effects are among the most intriguing neurofeedback effects, and are the most valued clinically, thus indicating the neurofeedback strength of inducing neuroplasticity [52], which has been validated to accrue two years post-neurofeedback intervention [103]. Although we can only assume that participants in our study were practicing the strategies they learned during the neurofeedback sessions at home, neurofeedback is based on endogenous plasticity, inducing self-organization, which is a product of the structure–function interplay and is suggested to underlie ongoing improvement [52]. Indeed, one of the neurofeedback therapeutic principals is the plastic homeostatic adaptation [104], which enables lasting brain tuning and supports functional reorganization towards normalizing neural activity. The latter, in turn, may be the cornerstone for the long-term behavioral improvements coupled in lasting neuroplasticity [52,103]. Thus, the neurofeedback therapy may induce a long-term change in neural activity, ameliorating anxiety and life satisfaction in adults with SOR, as this study demonstrated at the one-month follow-up.

Importantly, being a feasibility study not using a control group, our findings are restricted in ruling out other factors that could have impacted the findings [51]; hence, our interpretation of findings should be cautiously regarded. Thus, this study requires further validation via a sham control trial [51]. Future studies should comprise a larger sample size and follow the CRED-nf checklist [51]. As a feasibility study, this study’s findings are encouraging, and may direct future randomized control trials in validating our results.

## 5. Conclusions

To conclude, this feasibility study is a first step in testing neuromodulation in individuals with SOR, utilizing neurofeedback. Of note, while studying the effect of upregulating the alpha band activity, it was the only frequency band with no change at follow-up—thus, conclusions are compromised. At the same time, findings may allude to future studies embracing a different perspective for alpha oscillation analysis, or targeting different oscillatory bands. Furthermore, findings indicate brain activity change only in the frontal region, which may partially explain the therapeutic gains found behaviorally via self-reports. Thus, the present study may suggest that neurofeedback holds merit as a therapeutic approach for adults with SOR, warranting future testing with a control group.

## Figures and Tables

**Table 1:** Sample characteristics (mean, SD, percentage) (N = 10).

Characteristics		Mean	SD	%
Age		33.11	6.47	
SRQ-IS	Hedonic	1.63	0.37	
	Aversive	2.86	0.27	
Education	University			55.6
	College			22.2
	Post-graduate			22.2

SD—standard deviation; SRQ—sensory responsiveness questionnaire; SRQ hedonic cutoff—2.76; SRQ aversive cutoff—2.39.

**Table 2:** Frontal electrodes power (mean (SD)) in the brain oscillatory bands at the four measurements (T1–T4) and intervention effects.

Bands	T1	T2	T3	T4	RMA Significance		T1 *vs.* T3 Comparison	T2 *vs.* T3 Comparison	T1 *vs.* T4 Comparison	T2 *vs.* T4 Comparison	T3 *vs.* T4 Comparison	T2 *vs.* T3 Cohen’s *d*	T2 *vs.* T4 Cohen’s *d*
					*F*	*p*	*p*	*p*	*p*	*p*	*p*		
Delta (1–4 Hz)	16.06 (9.02)	19.36 (17.48)	16.08 (9.75)	25.19 (15.21)	4.56	0.01	>0.05	>0.05	0.019	>0.05	0.019	0.29	0.801
Theta (4–8 Hz)	5.08 (3.00)	4.87 (2.43)	5.38 (3.16)	8.40 (3.65)	7.12	<0.001	>0.05	>0.05	0.005	0.003	0.012	0.51	1.126
Alpha (8–12 Hz)	11.93 (9.44)	11.60 (8.11)	12.27 (10.31)	12.03 (7.60)		>0.05						0.43	0.14
Beta (12–30 Hz)	2.28 (1.06)	2.25 (0.80)	2.30 (1.06)	5.82 (5.16)	3.63	0.02	>0.05	>0.05	0.056	0.064	0.058	0.42	0.684
Gamma (>30 Hz)	0.16 (0.04)	0.14 (0.05)	0.15 (0.07)	1.42 (2.05)	3.32	0.03	>0.05	>0.05	0.073	0.084	0.071	0.019	0.637

T1—baseline; T2—pre-intervention; T3—post-intervention; T4—follow-up; SD—standard deviation; Hz—hertz; RMA—repeated measures ANOVA; *p* values higher than 0.09 are presented as >0.05.

**Table 3:** Mean (SD), median, and IRQ of study measures (PSQ, SWLS, GAS, STAI) in the four measurements (T1–T4).

Measurement		Score Range	Time 1			Time 2			Time 3			Time 4		
			Mean (SD)	MED	IRQ	Mean (SD)	MED	IRQ	Mean (SD)	MED	IRQ	Mean (SD)	MED	IRQ
PSQ	Total *	0–10	5.45 (1.90)	5.46	3.87–7.01	5.90 (2.13)	6.00	4.46–7.51	5.36 (2.03)	5.42	3.89–6.96	5.31 (2.00)	5.14	3.89–6.69
	Minor	0–10	4.84 (1.89)	4.64	3.39–6.32	5.05 (2.19)	5.14	3.25–6.42	4.40 (1.82)	4.42	3.00–5.71	4.35 (1.88)	4.42	2.92–5.21
	Moderate	0–10	6.05 (2.00)	6.14	4.35–7.71	6.74 (2.24)	7.14	5.03–8.32	6.32 (2.38)	7.00	3.82–8.42	6.27 (2.32)	6.50	4.50–8.10
SWLS ^		5–35	21.10 (6.26)	19.25	17.25–26	20.40 (7.07)	22.50	14.50–24.50	22.10 (7.43)	23.00	15.00–27.00	23.30 (7.11)	24.50	17.75–29.00
GAS *^,^^		−2–+2	−2 (0.0)	−2	−2–−2	−1.85 (0.33)	−2	−2–−1.87	−0.15 (0.68)	−0.25	−1 −0.62	−0.40 (1.32)	−0.5	−1.62–0.62
STAI	State	20–80	45.4 (1.90)	5.46	40.75–48.5	46.40 (4.92)	45.50	4300.−47.25	47.20 (3.35)	47.50	44.75–50.25	46.50 (4.94)	48.00	48–50
	Trait	20–80	46.40 (4.29)	47.00	41.75–50.25	45.90 (5.08)	46.50	40.75–49.50	44.30 (4.94)	44.00	39.75–47.00	43.80 (3.52)	43.50	40.75–46.25

PSQ—pain sensitivity questionnaire; SWLS—satisfaction with life scale; GAS—goal attainment scaling; STAI—state–trait anxiety inventory; T1—baseline; T2—pre-intervention; T3—post-intervention; T4—follow-up; SD—standard deviation; MED—median; IQR—interquartile range; *—denotes statistically significant differences (*p* < 0.05) between T2 and T3; ^—denotes statistically significant differences (*p* < 0.05) between T2 and T4.

## Data Availability

The data presented in this study are available on request from the corresponding author.

## Appendix A

**Table A1:** CRED-nf best practices checklist 2020.

Domain	Item #	Checklist Item	Reported on Page #
Re-experiment			
	1a	Pre-register experimental protocol and planned analyses	p. 3
	1b	Justify sample size Control groups	p. 4
Control groups			
	2a	Employ control group(s) or control condition(s)	Control condition: T1 p. 3
	2b	When leveraging experimental designs where a double-blind is possible, use a double-blind	
	2c	Blind those who rate the outcomes, and when possible, the statisticians involved	p. 3
	2d	Examine to what extent participants and experimenters remain blinded	
	2e	In clinical efficacy studies, employ a standard-of-care intervention group as a benchmark for improvement	
Control measures			
	3a	Collect data on psychosocial factors	p. 7; p. 5
	3b	Report whether participants were provided with a strategy	p. 6
	3c	Report the strategies participants used	
	3d	Report methods used for online-data processing and artefact correction	p. 4
	3e	Report condition and group effects for artefacts	
Feedback specifications			
	4a	Report how the online-feature extraction was defined	
	4b	Report and justify the reinforcement schedule	p. 6
	4c	Report the feedback modality and content	p. 6
	4d	Collect and report all brain activity variable(s) and/or contrasts used for feedback, as displayed to experimental participants	p. 6
	4e	Report the hardware and software used	p. 6.
Outcome measures			
pre-experiment	5a	Report neurofeedback regulation success based on the feedback signal	p. 9
	5b	Plot within-session and between-session regulation blocks of feedback variable(s), as well as pre-to-post resting baselines or contrasts	p. 8
	5c	Statistically compare the experimental condition/group to the control condition(s)/group(s) (not only each group to baseline measures)	Table 2, Table 3
pre-experiment	6a	Include measures of clinical or behavioural significance, defined a priori, and describe whether they were reached	p. 9
	6b	Run correlational analyses between regulation success and behavioural outcomes	p. 10
Data Storage			
	7a	Upload all materials, analysis scripts, code, and raw data used for analyses, as well as final values, to an open access data repository, when feasible	
